# Laparoscopic treatment of heterotopic pancreas in the prepyloric region

**DOI:** 10.4103/0972-9941.28186

**Published:** 2006-12

**Authors:** R Gálvez-Valdovinos, A Mendoza-Rodríguez, J H Coronado-Perez, E Marin Santillan, F Funes-Rodríguez

**Affiliations:** Departments of Surgery and Endoscopy, “Hospital Angeles”, León Guanajuato, México

**Keywords:** Ectopic pancreas, heterotopic pancreas, laparoscopy

## Abstract

Heterotopic pancreas is a rare condition and its diagnosis is often difficult. Traditionally the condition is treated by open surgery. We report two young women with symptomatic heterotopic pancreas located in the prepyloric region. In the first patient, upper gastrointestinal endoscopy identified a round sessile lesion with a central umbilication of the mucosa without bleeding and in the second endoscopy showed a lesion with intraluminal protrusion. In both cases, a diagnostic laparoscopy identified masses amenable to laparoscopic excision. Intraoperative histology confirmed ectopic pancreatic tissue in both. In the treatment of heterotopic pancreas, laparoscopic excision provides a feasible, safe and effective treatment option.

## INTRODUCTION

Heterotopic pancreas (HP) is defined as pancreatic tissue found outside the ectopic pancreas without any anatomic or vascular connections between them.[1] Gastric antrum and prepyloric region form common sites for HP. The gastric heterotopic pancreas is often asymptomatic. Rarely, it may cause recurrent epigastric pain or manifest with upper gastrointestinal bleeding. Treatment is required for symptomatic gastric HP and lesions larger than 3 cm in size.[2] Laparoscopic excision forms an attractive treatment option in these patients, the extent of which is generally guided by an intraoperative histologic examination.[3] For asymptomatic small lesions less than 2 cm, follow-up may be considered after histopathological confirmation of their benign nature.

We report two patients with chronic recurrent abdominal pain and upper gastrointestinal bleeding caused by HP and their successful laparoscopic management.

## CASE REPORTS

### Case 1

**Figure 1:**
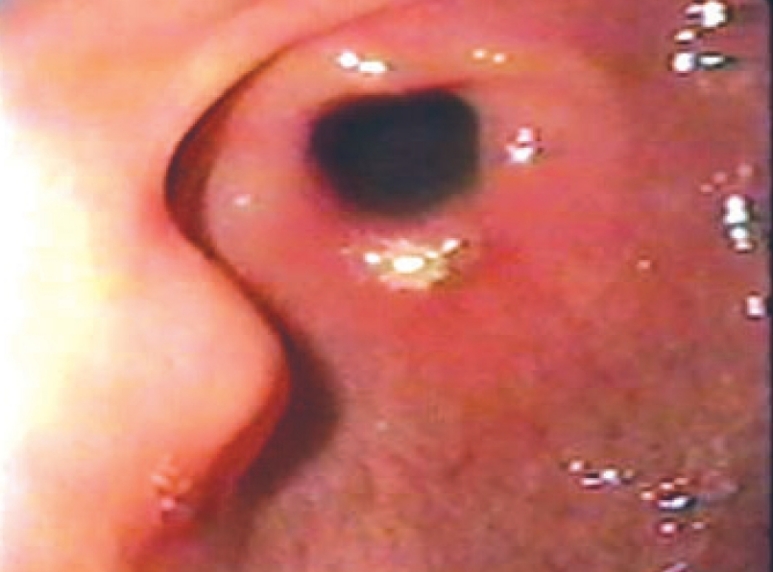
The endoscopic study showed a round sessile nodule with a central umbilication of the mucosa

A 28-year-old woman with past history of recurrent attacks of epigastric pain and progressive dyspeptic symptoms was admitted with a bout of hematemesis. General and abdominal examinations were unremarkable. Blood count and liver function tests were normal. An upper gastrointestinal endoscopy revealed a nonbleeding, firm, round sessile nodular lesion with central umbilication in the gastric antrum [Figure 1]. A surface biopsy revealed normal mucosa. Abdominal computerized tomography was normal. The patient was offered laparoscopic surgery, which was performed under general anesthesia with endotracheal intubation. A nasogastric tube and indwelling urinary catheter were inserted. The patient was placed in a lithotomy position and the operation table tilted head-up by 30°. Pneumoperitoneum was established and a 10 mm port was inserted 5 cm above the umbilicus in the midline. Two additional ports were inserted under direct vision - a 10 mm port in the left and 5 mm port in the right subcostal area. Laparoscopy revealed a 2 cm mass in the prepyloric area 3 cm proximal to the pylorus. A laparoscopic excision was performed. An intraoperative histology study revealed ectopic pancreatic tissue. The postoperative course was uneventful and the patient was discharged 4 days after surgery. The patient remains asymptomatic 60 months after surgery and a follow-up endoscopy study was normal.

### Case 2

**Figure 2:**
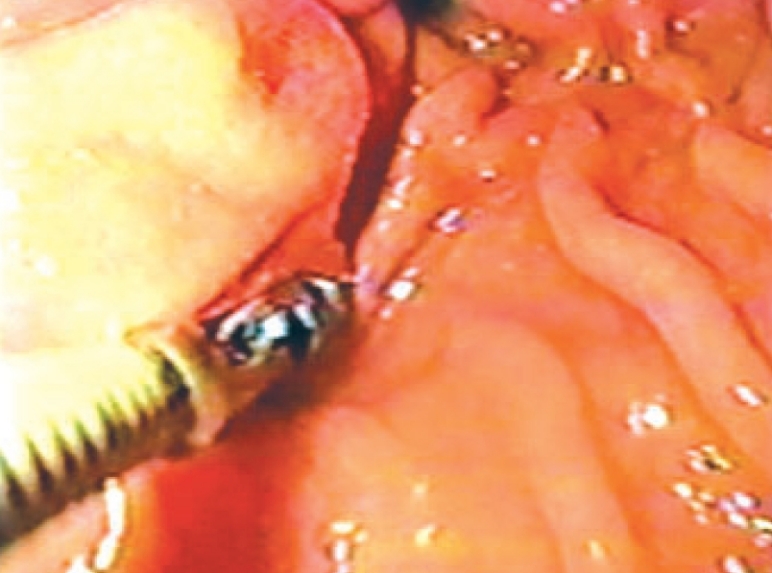
An esophagogastroduodenoscopy revealed an intraluminal protrusion in the prepyloric area

**Figure 3:**
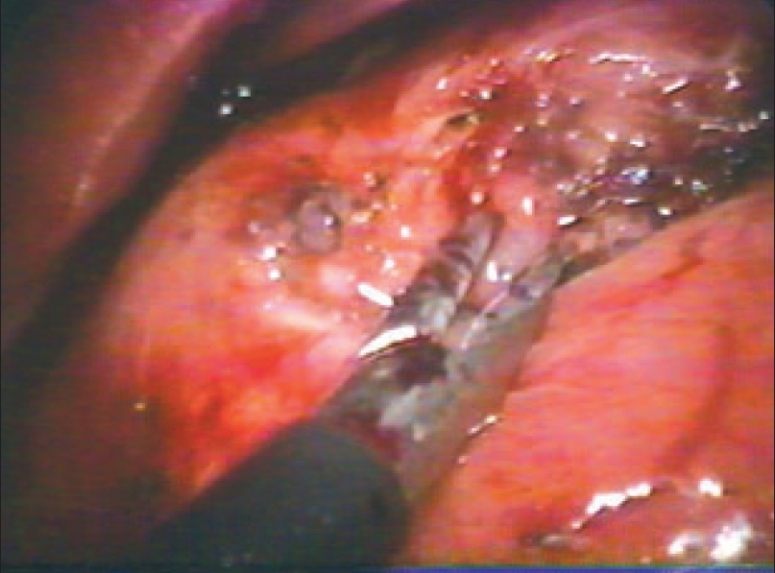
Laparoscopic resection

A 32-year-old female with a 2 year history of recurrent upper abdominal pain was admitted with progressively increasing epigastric pain radiating laterally. She was in good health and her vital signs, cardiovascular and respiratory systems were normal. Abdominal palpation revealed epigastric tenderness. Blood count, liver function tests, amylase and lipase tests were normal. Abdominal ultrasound and computerized tomography were unremarkable. An upper gastrointestinal endoscopy revealed an intraluminal protrusion in the prepyloric area [Figure 2]; the mucosa over the mass was normal both visually and on biopsy. An exploratory laparoscopy revealed a solid 2.5 cm mass located in the anterior wall of the gastric antrum within 2 cm of the pyloric canal. A laparoscopic excision was performed. An intraoperative frozen section showed gastric glands and multiple ducts and islands of pancreatic tissue [Figure 3]. The gastric defect was closed by intracorporeal interrupted nonabsorbable sutures reinforced by an omental patch. Her recovery was uneventful and postoperative water-soluble contrast media study was normal. The patient was discharged 5 days after surgery and remains asymptomatic during the 48 months of follow-up period. The last upper gastrointestinal endoscopy was normal.

## DISCUSSION

**Figure 4:**
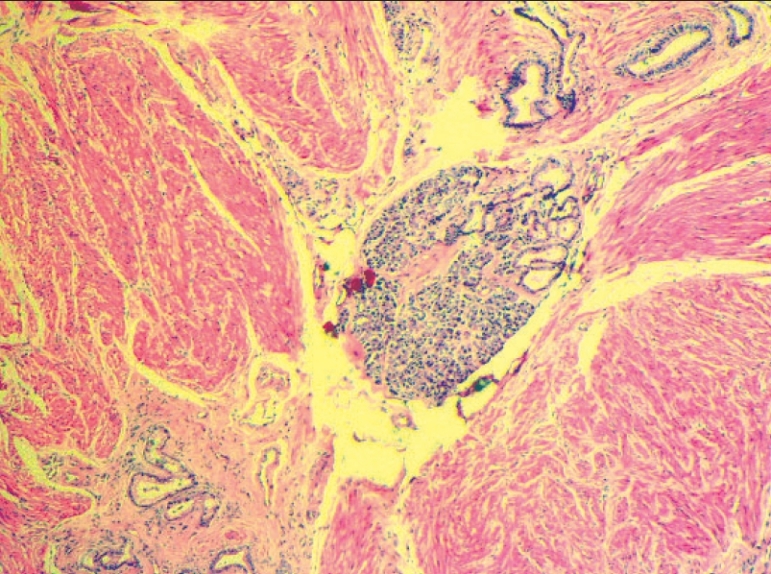
Histologic: Type I in bouth cases

The estimated incidence of HP is 1 per 500 upper abdominal operations and between 0.6 and 14.0% in autopsy cases.[1,2] More frequently, these lesions are detected accidentally during routine endoscopic study or exploratory laparotomy. Gastric lesions are located in the gastric antrum (85 to 95%), along the greater curvature of the stomach or in the prepyloric area.[3] HP is a congenital disorder that consists in the presence of normal pancreatic tissue located outside the pancreatic frame.[4] It originates from the submucosa in two-thirds of the cases and in the remainder from the muscularis mucosa or the subserosal layer.[3] Histologically, these lesions have been classified into three categories depending on the degree of resemblance to normal pancreatic tissue. The most common group (type I) is the pancreatic tissue that is easily identified with ducts, acini and endocrine islets [Figure 4]. In the second group (type II) there are a few acini and many ducts, while in the last common category (type III) only ducts are seen. Smooth muscle is commonly found throughout these lesions.[5]

HP often remains asymptomatic throughout life but may sometimes cause symptoms including recurrent epigastric pain, upper gastrointestinal bleeding, gastric ulceration, gastric outlet obstruction or pancreatitis. In a few cases, complications such as pancreatic pseudocyst or cyst formation supervene. Association with insulinoma, adenoma and malignant transformation has been reported.[2] All benign or malignant pathologic processes described in the pancreas may be expected to occur in ectopic pancreas.[1]

The differential diagnosis includes a number of benign and malignant gastric wall tumors, intramural vessels and extrinsic compression from extramural structures.[2] The classic endoscopic appearance is that of a small well-circumscribed submucosal protrusion with a normal overlying mucosa.[3] In less than half the cases, umbilication may be present; this may be the site of ductal drainage to the mucosal surface. Usually these lesions are firm, round sessile nodules bulging into the lumen and can bleed with endoscopic manipulation.[2] The characteristic radiographic appearance of HP in the stomach has been described as a small broad-based submucosal mass in the antrum, with a central umbilication that represents a rudimentary pancreatic duct; the mass resembles leiomyoma or other submucosal tumors such as carcinoid or intramural metastasis.[4] Once in a while, the HP appears as a mass with an irregular surface indistinguishable from an adenomatous polyp or a polypoid carcinoma.[5]

As can be seen from the case reports of both our patients, HP are eminently suitable for laparoscopic excision if situated in a suitable location. An intraoperative biopsy is recommended in order to prevent unnecessary extensive surgery. The application of laparoscopic surgery to HP in the stomach appears to be a technically feasible, safe and effective treatment for symptomatic patients. It should be considered a viable alternative to open surgery.
